# Long-term Effectiveness Associated With the BNT162b2 Vaccine Against SARS-CoV-2 Infection Among Adolescents in South Korea

**DOI:** 10.1001/jamanetworkopen.2022.27205

**Published:** 2022-08-17

**Authors:** Jia Kim, Young June Choe, Hyunju Lee, Eun Hwa Choi, Eun Jung Jang, Ryu Kyung Kim, Young-Joon Park

**Affiliations:** 1 Korea Disease Control and Prevention Agency, Cheongju, South Korea; 2 Department of Pediatrics, Korea University Anam Hospital, Seoul, South Korea; 3 Department of Pediatrics, Seoul National University College of Medicine, Seoul, South Korea

## Abstract

This cohort study estimates the effectiveness associated with the BNT162b2 vaccine against infection and critical infection of SARS-CoV-2 among adolescents in South Korea.

## Introduction

The emergence of the SARS-CoV-2 Omicron variant has resulted in a surge of COVID-19 cases among adolescents.^1^ We estimated the effectiveness associated with the BNT162b2 vaccine against SARS-CoV-2 infection and critical infection among adolescents in South Korea.

## Methods

In this cohort study, we compared the rates of SARS-CoV-2 infection and critical infection by age, census region, vaccination status, and vaccine doses in all adolescents aged 12 to 18 years in South Korea between July 19, 2021, and January 22, 2022. Infection was defined as symptomatic or asymptomatic SARS-CoV-2 positivity as confirmed by polymerase chain reaction and/or rapid antigen testing. Critical infection was defined as hospitalization for COVID-19 with high-flow oxygen therapy, mechanical ventilation, extracorporeal membrane oxygenation, or continuous kidney replacement therapy within 28 days of laboratory confirmation of SARS-CoV-2 positivity. A time-dependent Cox proportional hazards regression model was used, and hazard ratios (HRs) with 95% CIs from an adjusted model with covariates (sex, age, days elapsed since vaccination, census region, immunocompromised status) were calculated to estimate vaccine effectiveness as (1 − HR) × 100. Person-days comprised the number of follow-up days of adolescents who were never vaccinated and days contributed by adolescents before being vaccinated or censored (eFigures 1 and 2 in the Supplement).

This study was conducted as a legally mandated public health investigation under the authority of the Korean Infectious Diseases Control and Prevention Act and thus did not require institutional review board approval. Informed consent was waived owing to the retrospective nature of the study. The study followed the STROBE reporting guideline.

## Results

Between July 19, 2021, and January 22, 2022, of the 3 203 985 adolescents included in the study (1 549 490 female [48.4%], 1 654 495 male [51.6%]), 57.4% were aged 12 to 15 years, 27.6% were aged 16 to 17 years, and 15.0% were aged 18 years (Table). Of these, 29 285 had SARS-CoV-2 infection and 11 had critical infection. Among adolescents with infection, 55.4% were unvaccinated, whereas 17.3% and 24.0% had received 1 and 2 doses, respectively. All 11 adolescents with critical infection were unvaccinated. The estimated effectiveness after 2 doses of BNT162b2 was 75.5% (95% CI, 65.8-82.4) among those aged 18 years, 80.4% (95% CI, 77.8-82.7) among those aged 16-17 years, and 79.2% (95% CI, 77.4-80.1) among those aged 12 to 15 years. The estimated effectiveness among those aged 18 years increased to 55.2% (95% CI, 47.3-61.9) 30 to 59 days after administration of a third dose. The effectiveness associated with the vaccine against infection and critical infection is shown in the Figure).

**Table.** Characteristics of Study Cohort^a^

Characteristic	Total	SARS-CoV-2 infection	
		Infection	Critical infection
Total	3 203 985	29 285	11
Age, y			
12-15	1 839 053 (57.4)	17 372 (59.3)	2 (18.2)
16-17	885 545 (27.6)	7384 (25.2)	7 (63.6)
18	479 387 (15.0)	4529 (15.5)	2 (18.2)
Sex			
Female	1 549 490 (48.4)	12 586 (43.0)	2 (18.2)
Male	1 654 495 (51.6)	16 699 (57.0)	9 (81.8)
Census region			
Urban	1 592 420 (49.7)	20 785 (71.0)	8 (72.7)
Rural	1 611 565 (50.3)	8500 (29.0)	3 (27.3)
Vaccination status			
Unvaccinated	3 203 985	16 220 (55.4)	11 (100.0)
Vaccinated			
1 Dose	2 607 319 (81.4)	5052 (17.3)	0
2 Doses, d	2 387 597 (74.5)	7041 (24.0)	0
0-13	2 387 597 (74.5)	939 (3.2)	0
14-29	1 988 027 (62.0)	860 (2.9)	0
30-59	1 674 505 (52.3)	2303 (7.9)	0
60-89	847 109 (26.4)	856 (2.9)	0
≥90	442 223 (13.8)	2083 (7.1)	0
3 Doses, d	288 024 (9.0)	972 (3.3)	0
0-13	288 024 (9.0)	237 (0.8)	0
14-29	233 850 (7.3)	488 (1.7)	0
30-59	140 243 (4.4)	247 (0.8)	0

^a^ Data are presented as No. (%) of study participants. Time-varying vaccine effectiveness was calculated at 14 days after the first dose; 0 to 13 days, 14 to 29 days, 30 to 59 days, and 60 to 89 days after the second dose; and 0 to 13 days, 14 to 29 days, and 30 to 59 days after the third dose. Because vaccination initiation was different between age groups, the observed time differed between adolescents aged 12 to 15 years (up to 30-59 days after the second dose), 16 to 17 years (up to 60-89 days after the second dose), and 18 years (up to 30-59 days after the third dose).

**Figure.**
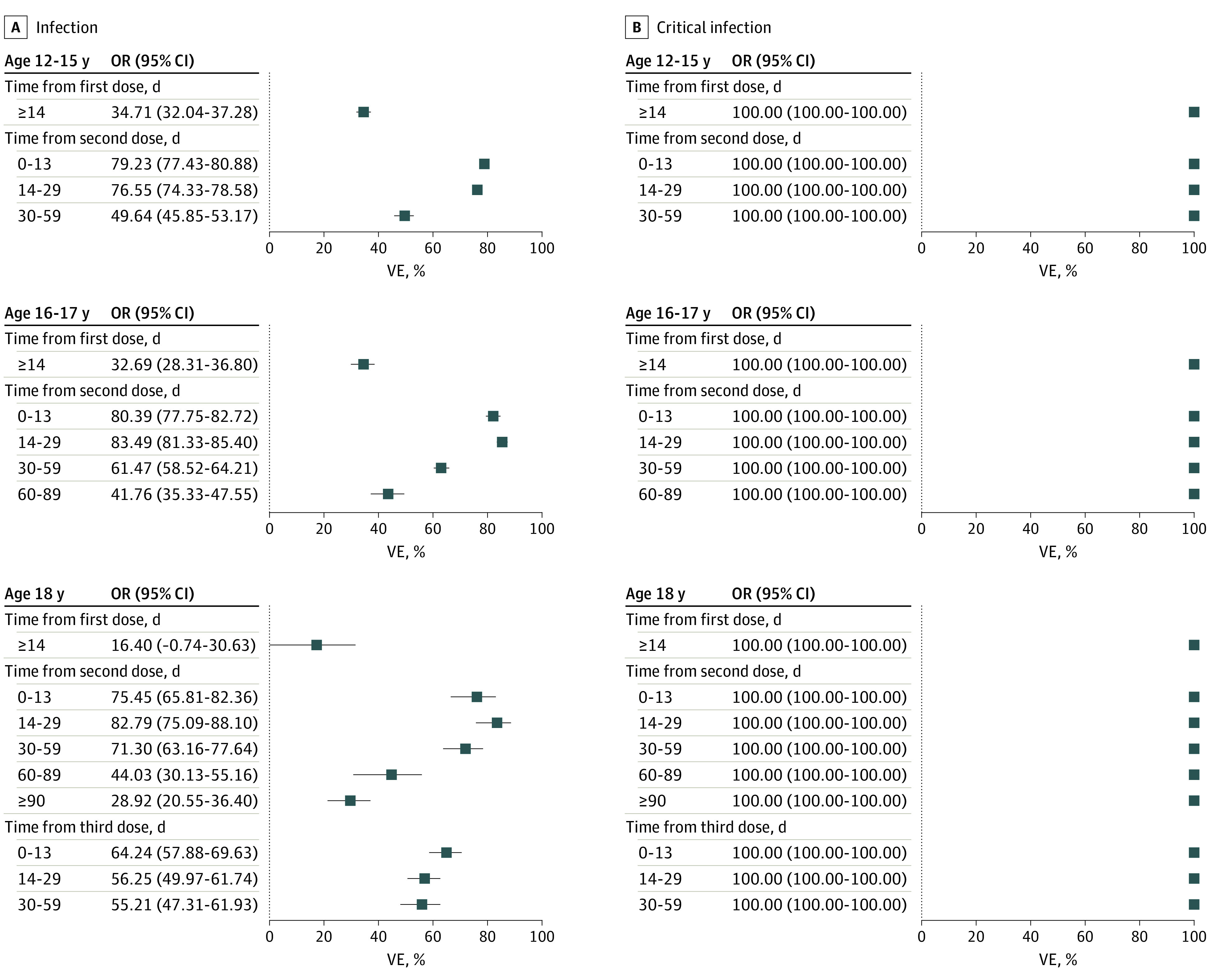
Vaccine Effectiveness (VE) Associated With BNT162b2 Against SARS-CoV-2 Infection and Critical Infection

## Discussion

Previous studies have shown decreased vaccine effectiveness after emergence of the SARS-CoV-2 Omicron variant.^2^ However, few studies have addressed the association between vaccination among adolescents and COVID-19 incidence. Our results suggest that after vaccination with BNT162b2, the effectiveness of the vaccine against SARS-CoV-2 infection among adolescents waned over time, with limited protection observed 30 to 59 days after administration of a second dose of the vaccine. Regardless, our findings suggest an association between the BNT162b2 vaccine and sustained effectiveness against critical SARS-CoV-2 infection, as reported previously.^3,4,5^

This study has some limitations. First, the difference in testing behavior based on vaccination status may have introduced bias, especially during the Omicron surge. Second, both SARS-CoV-2 genomic variance and the timing of vaccination may have affected differences in vaccine effectiveness among the study participants.

Our findings suggest that 2 doses of the BNT162b2 vaccine may be sufficient for protection against critical SARS-CoV-2 infection among adolescents, albeit with waning immunity. Furthermore, booster vaccinations may be required for continued protection among adolescents.

## Notes

Supplement. **eFigure 1.** Observation Period and Data Censored in Adolescents Aged 12 to 18 Years, South Korea **eFigure 2.** Inclusion Criteria of COVID-19 Vaccine Effectiveness in Adolescent Cohort Study, South Korea
