# Supplementary material for: Long-term Effectiveness Associated With the BNT162b2 Vaccine Against SARS-CoV-2 Infection Among Adolescents in South Korea
Source: JAMA Netw Open. 2022 Aug 17;5(8):e2227205. doi: 10.1001/jamanetworkopen.2022.27205 (PMC9386535; doi:10.1001/jamanetworkopen.2022.27205)
Supplement: Supplement. — eFigure 1. Observation Period and Data Censored in Adolescents Aged 12 to 18 Years, South Korea eFigure 2. Inclusion Criteria of COVID-19 Vaccine Effectiveness in Adolescent Cohort Study, South Korea [file jamanetwopen-e2227205-s001.pdf]

Supplemental Online Content

Kim J, Choe YJ, Lee H, et al. Estimated long-term effectiveness associated with the BNT162b2 vaccine against SARS-CoV-2 infection among adolescents in South Korea. *JAMA Netw Open*. 2022;5(8):e2227205. doi:10.1001/jamanetworkopen.2022.27205

eFigure 1. Observation Period and Data Censored in Adolescents Aged 12 to 18 Years, South Korea
eFigure 2. Inclusion Criteria of COVID-19 Vaccine Effectiveness in Adolescent Cohort Study, South Korea

This supplemental material has been provided by the authors to give readers additional information about their work.

© 2022 Kim J et al. *JAMA Network Open.*

eFigure 1. Observation Period and Data Censored in Adolescents Aged 12 to 18 Years, South Korea

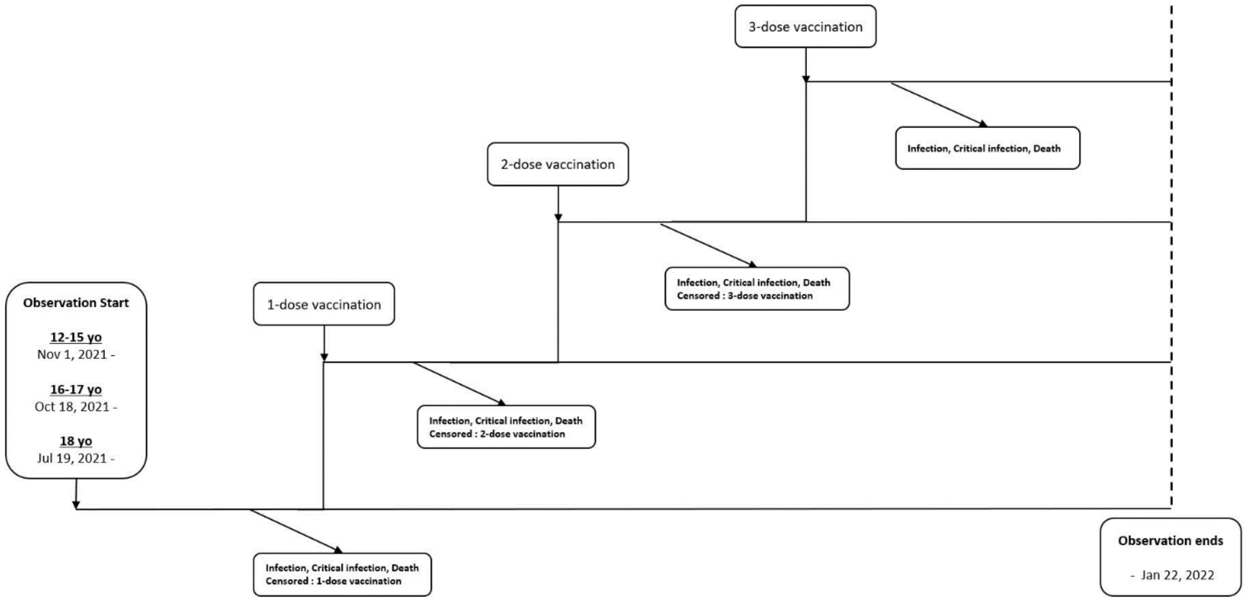

© 2022 Kim J et al. *JAMA Network Open.*

eFigure 2. Inclusion Criteria of COVID-19 Vaccine Effectiveness in Adolescent Cohort Study, South Korea

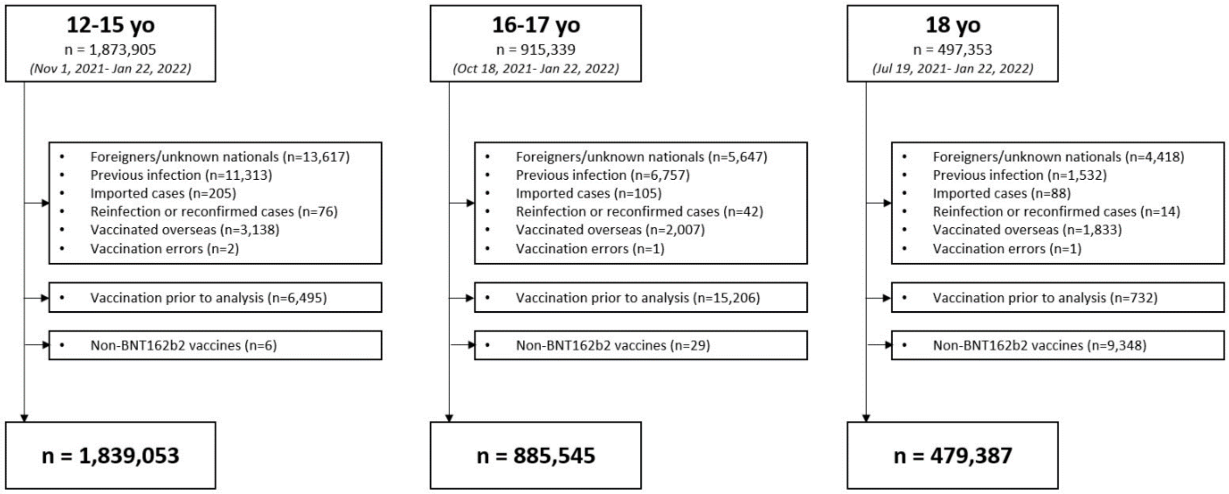

© 2022 Kim J et al. *JAMA Network Open.*
